# Efficacy and safety of Bifidobacterium quadruple viable tablets combined with mosapride citrate in the treatment of constipation in China: a systematic review and meta-analysis

**DOI:** 10.1186/s12876-023-02884-3

**Published:** 2023-07-18

**Authors:** Mei Luo, Lishou Xiong, Lu Zhang, Qinchang Xu

**Affiliations:** 1 Department of Gastroenterology and Hepatology, The First Affiliated Hospital of Sun Yat-Sen University, NO.58 Zhongshan Road 2, 510080 Guangzhou, China; 2 Hangzhou Grand Biologics Pharmaceutical Co. LTD, Hangzhou, 050000 China

**Keywords:** Bifidobacterium, Functional constipation, Efficacy, Mosapride citrate, meta-analysis

## Abstract

**Aim:**

To analyze the efficacy and safety of Bifidobacterium quadruple viable tablets combined with mosapride citrate for the treatment of constipation.

**Methods:**

A systematic review was performed on studies published until July 2022 in PubMed, Embase, China National Knowledge Infrastructure, and Wanfang. The efficacy rate, adverse reaction rate, recurrence rate, and clinical symptoms were included in the measured outcomes.

**Results:**

The efficacy of Bifidobacterium quadruple viable tablets combined with mosapride citrate in the treatment of constipation was higher than that of mosapride citrate alone (OR = 4.75, 95% CI (3.27, 6.90), Z = 8.19, *P* < 0.001; I^2^ = 0.0%, *P* = 0.645). There was no significant difference in the incidence of adverse reactions between the two groups (OR = 0.97, 95% CI (0.61,1.57), Z = 0.11, *P* = 0.911; I^2^ = 0.0%, *P* = 0.958). The recurrence rate of constipation in patients receiving the combination treatment was lower than that of patients treated with mosapride citrate alone (OR = 0.48, 95%CI (0.31, 0.73), Z = 3.38, *P* = 0.001; I^2^ = 29.8%, *P* = 0.200).

**Conclusions:**

Bifidobacterium quadruple viable tablets combined with mosapride citrate demonstrated efficacy and safety in treating constipation. Probiotics have the potential to positively influence gut health and microbial profiles in patients with functional constipation.

**Supplementary Information:**

The online version contains supplementary material available at 10.1186/s12876-023-02884-3.

## Introduction

Functional constipation (FC), also referred to as chronic idiopathic constipation, is a prevalent gastrointestinal disorder caused by abnormal functioning of the colon, rectum and anus [1]. Patients with FC usually present with symptoms such as hard or lumpy stools, reduced frequency of defecation, a sensation of incomplete evacuation or blockage, and straining at defecation. Additionally, some patients also report abdominal pain and bloating. In general, symptoms are deemed to be chronic if they have been present for at least 3 months [2, 3]. In recent years, there has been a rise in the incidence of FC due to lifestyle and dietary changes. Globally, the prevalence of FC has been reported to range from 1.9 to 27.2%, with an average of approximately 14.8% [4]. FC negatively impacts patients, seriously affecting their quality of life [5]. Constipation has been associated with a greater than two-fold risk of colon cancer [6]. Furthermore, constipation tends to increase with age and often coexists with cardiovascular risk factors [7].

Fiber and osmotic laxatives are usually as the first-line treatment for FC. However, recurrence of symptoms is often observed following this treatment. Recently, there has been an increase in the use of gastrointestinal motility drugs, such as mosapride citrate (mosapride), in clinical practice. Mosapride is a selective 5-hydroxytryptamine 4 (5-HT4) receptor agonist that promotes the release of acetylcholine and enhances the peristaltic function of the gastrointestinal tract by activating cholinergic interneurons and 5-HT4 receptors in the muscular plexus [8, 9]. Mosapride has demonstrated effectiveness in patients with constipation-type irritable bowel syndrome, reducing bowel transit time and decreasing bowel flatus [10]. Currently, clinical scholars believe that patients with FC exhibit reduced levels of obligate anaerobic bacteria, intestinal dysfunction, imbalanced intestinal flora, increased intestinal pH, and slow intestinal peristalsis [11]. Therefore, it is particularly important to restore the ecological balance of intestinal microbiota in these patients. Bifidobacterium quadruple viable tablets contain *Bifidobacterium*, *Enterococcus faecalis*, *Lactobacillus acidophilus*, and other components that can lower intestinal PH 4, promote the restoration of normal flora, enhance immune function, avoid the invasion of pathogenic bacteria, and facilitate the recovery of gastrointestinal motility [12–14]. In patients with FC, Bifidobacterium quadruple viable tablets can form an effective biological barrier, alleviating symptoms such as abdominal pain, diarrhea, and abdominal distension, while promoting the adjustment of intestinal flora and restoration of normal intestinal function, all with a high level of safety [15].

The efficacy of a single drug is often limited, and prolonged use can lead to drug resistance and dependence, affecting the compliance of patients to treatment and the long-term effectiveness of the drug. Although Bifidobacterium quadruple viable tablets regulate human microecology and have a notable impact on the intestine, they do not affect gastrointestinal motility. In contrast, mosapride can increase gastrointestinal motility but cannot effectively improve the intestinal microenvironment. In recent years, combining these two drugs has shown improved therapeutic outcomes in patients with FC [16]. However, there is a notable lack of systematic reviews and meta-analyses investigating the combined efficacy and safety of these two drugs as a treatment for FC. To bridge this gap in knowledge, we conducted a comprehensive meta-analysis to evaluate the efficacy and safety profile of this combined therapy in managing FC.

## Methods

### Literature search

The meta-analysis was conducted in accordance with the Preferred Reporting Items for Systematic reviews and Meta-Analyses (PRISMA) guidelines. A comprehensive search was performed by two reviewers in two English-language databases (PubMed and Embase) and two Chinese-language databases (China National Knowledge Infrastructure and Wanfang) from database inception through July 2022. The search terms used were as follows: (bifidobacterium), AND (functional constipation), AND (idiopathic constipation), AND (chronic constipation), AND (slow transit). The full text of the included articles was carefully reviewed.

### Inclusion and exclusion criteria

The inclusion criteria were as follows: (1) prospective or retrospective studies or clinical research; (2) enrollment of patients with constipation; (3) the control group receiving treatment with mosapride citrate tablets, and the experimental group receiving treatment with Bifidobacterium quadruple viable tablets on the basis of the control group; and (4) primary outcome measures focused on the effectiveness in the treatment of constipation, while secondary outcomes included adverse event rate, recurrence rate, and clinical symptoms. The following criteria resulted in the exclusion of identified articles: (1) duplicate publications; (2) conference summaries, comments, letters, existing meta-analyses, and systematic reviews; (3) non-randomized controlled trials; and (4) studies investigating abnormal index values.

### Data extraction

The following information was extracted from the included studies: author’s name(s), publication date, sample size, patient age, outcome indicators, and details of intervention and control group treatments (treatment type and method of administration). The first reviewer extracted data from the literature, and the second reviewer confirmed the accuracy.

### Statistical analysis

Statistical analyses were performed using Stata 15.0 software. The efficacy rate, adverse reaction rate, and recurrence rate of the two drug groups used to treat constipation were estimated using the odds ratio (OR) with a confidence interval (CI) of 95%. The clinical symptom scores were estimated using the Standardized Mean Difference (SMD) with a CI of 95%. A random effects model was utilized to estimate the overall effect of the two drug groups. Heterogeneity was estimated using the *I*^2^ test, and sensitivity analysis was conducted to evaluate the stability of the main indicators. Funnel plots and Egger’s and Begg’s statistics were utilized to examine publication bias. The risk of bias in the included studies was evaluated using the Cochrane Collaboration tool.

## Results

### Study selection

**Fig. 1:**
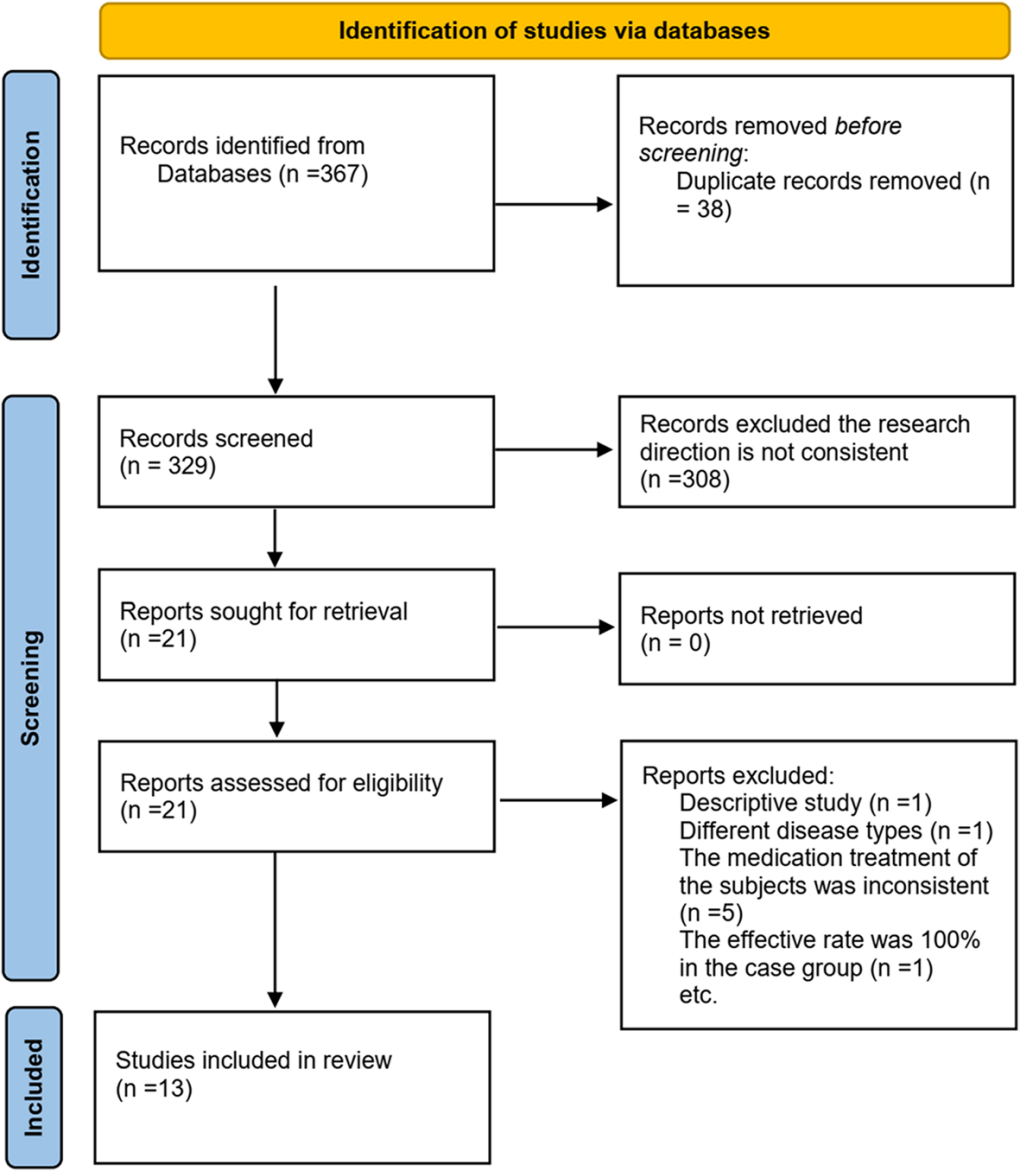
The process of selecting articles for the meta-analysis

**Fig. 2:**
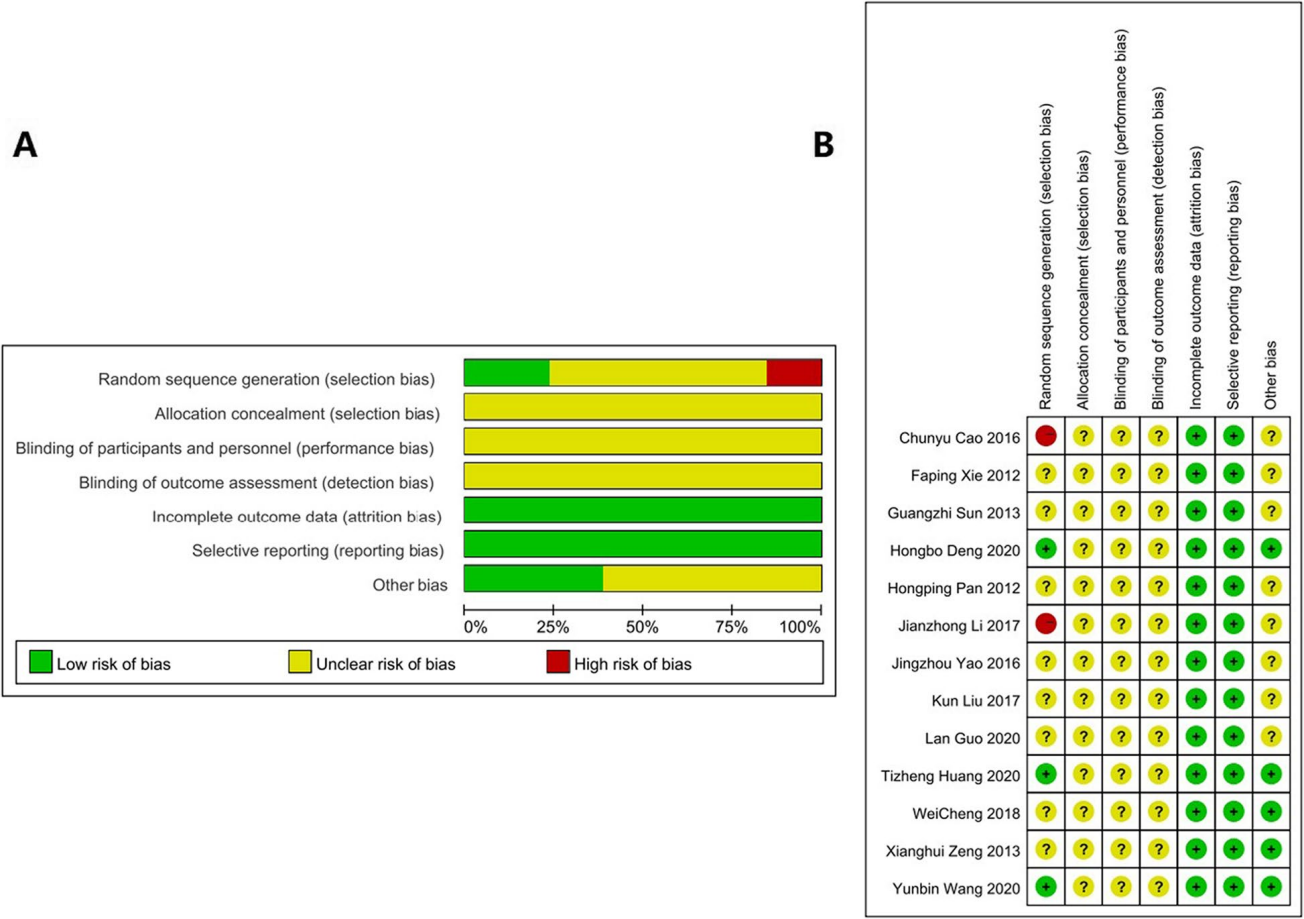
** A** Methodological quality evaluation of included studies. **B** Risk of bias summary

A total of 367 studies were identified through the database searches. Of these, 38 studies were duplicate studies and therefore excluded. Following screening of the titles and abstracts, 308 studies were deemed irrelevant and were also excluded. Based on the inclusion and exclusion criteria, 8 studies were excluded, leaving 13 studies that met the inclusion criteria (Fig. 1). The characteristics of the selected studies are presented in Table 1. The risk of bias evaluation results for the included studies are shown in Fig. 2A and B. Three studies described the randomization method, while blinded settings were not reported in any of the studies. Overall, we concluded that the included studies were of high quality with a low risk of bias.

**Table 1:** Basic information of the studies was included

Author name	Group	Samples	Age (years)	Intervention	Medicine	Outcomes
Huang et al. 2020 [30]	Treatment	50	NR	Control + Bifidobacterium quadruple viable tablets	1.5 g/once, tid,4 week	1, 3
	Control	50	NR	mosapride citrate	5 mg/once, tid,4 week	
Guo et al. 2020 [31]	Treatment	50	NR	Control + Bifidobacterium quadruple viable tablets	0.5 g/once,bid,4 week	1, 2
	Control	50	NR	mosapride citrate	5 mg/once, tid,4 week	
Li et al. 2017 [32]	Treatment	29	69.48 ± 5.26	Control + Bifidobacterium quadruple viable tablets	1.5 g/once, tid,4 week	1, 2, 4
	Control	29	69.38 ± 5.19	mosapride citrate	5 mg/once, tid,4 week	
Liu et al. 2017 [33]	Treatment	39	66.5 ± 1.5	Control + Bifidobacterium quadruple viable tablets	0.5 g/once,bid,8 week	1, 2, 3
	Control	39	67.3 ± 2.0	mosapride citrate	5 mg/once, tid,8 week	
Sun et al. 2013 [34]	Treatment	83	36.1 ± 6.0	Control + Bifidobacterium quadruple viable tablets	1.5 g/once, tid,4 week	1, 2, 3
	Control	83	35.4 ± 5.6	mosapride citrate	5 mg/once, tid,4 week	
Yao et al. 2016 [35]	Treatment	40	NR	Control + Bifidobacterium quadruple viable tablets	1.5 g/once, tid,4 week	3
	Control	40	NR	mosapride citrate	5 mg/once, tid,4 week	
Zeng et al. 2013 [36]	Treatment	47	41.7 ± 10.6	Control + Bifidobacterium quadruple viable tablets	1.5 g/once, tid,4 week	1, 2, 3
	Control	47	50.2 ± 7.8	mosapride citrate	5 mg/once, tid,4 week	
Xie et al. 2012 [37]	Treatment	48	NR	Control + Bifidobacterium quadruple viable tablets	1.5 g/once, tid,4 week	1, 2, 3
	Control	48	NR	mosapride citrate	5 mg/once, tid,4 week	
Pan et al. 2012 [38]	Treatment	40	52.7 ± 6.8	Control + Bifidobacterium quadruple viable tablets	0.5 g/once,tid,8 week	1, 2, 3
	Control	40	52.1 ± 7.1	mosapride citrate	5 mg/once,tid,8 week	
Cao et al. 2016 [39]	Treatment	64	67.82 ± 9.17	Control + Bifidobacterium quadruple viable tablets	0.5 g/once,bid,4 week	1, 2, 4
	Control	64	67.86 ± 4.21	mosapride citrate	5 mg/once,bid,4 week	
Deng et al. 2020 [40]	Treatment	45	70.5 ± 7.5	Control + Bifidobacterium quadruple viable tablets	0.5 g/once,bid,4 week	1, 2, 4
	Control	45	70.8 ± 8.1	mosapride citrate	5 mg/once,tid,4 week	
Cheng et al. 2018 [41]	Treatment	34	67.87 ± 4.22	Control + Bifidobacterium quadruple viable tablets	0.5 g/once,tid,4 week	2, 4
	Control	34	67.90 ± 4.18	mosapride citrate	5 mg/once,1once/day,4 week	
Wang et al. 2020 [42]	Treatment	52	40.5 ± 5.4	Control + Bifidobacterium quadruple viable tablets	1.5 g/once, tid, 12 week	1
	Control	52	39.6 ± 4.8	mosapride citrate	5 mg/once, tid, 12 week	

1 Effective rate, 2 Adverse reaction rate, 3 Recurrence rate, 4 clinical symptoms, *bid *Twice a day, *tid *Three times a day. *NR *Not reported

### Efficacy rate of treatment

**Fig. 3:**
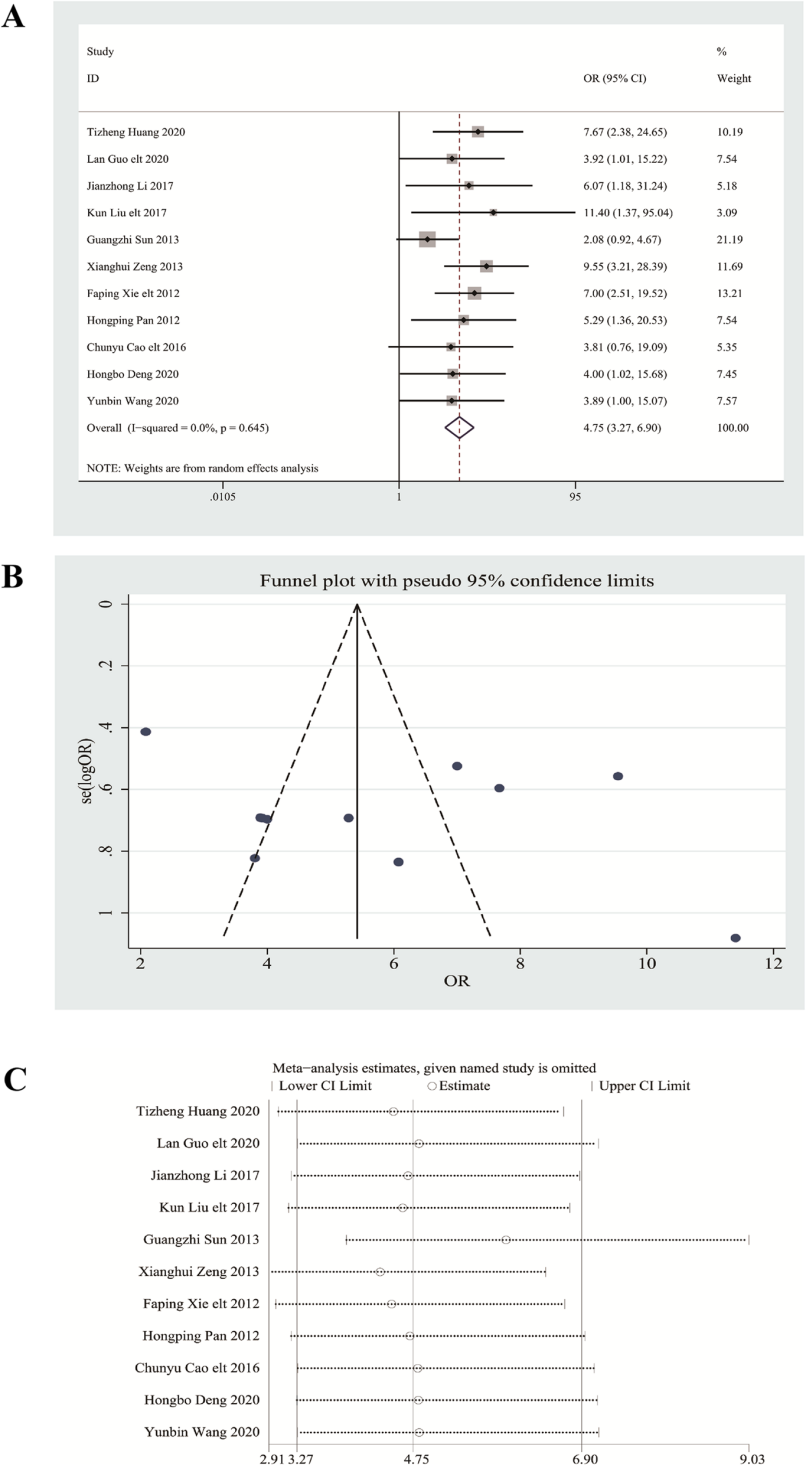
** A** The combined effect results of effective rate in each study. **B** Analysis of publication bias with the funnel plot about the effective rate. **C** Sensitivity analysis of effective rate

Of the 13 selected articles, 11 analyzed the effectiveness rate of treatments. The results of the random effects model revealed that the combination of Bifidobacterium quadruple viable tablets with mosapride citrate exhibited a higher efficacy rate in treating constipation than that of mosapride citrate tablets alone (OR = 4.75, 95% CI (3.27, 6.90), Z = 8.19, *P* < 0.001) (Fig. 3A). No heterogeneity was observed among the studies (*I*^2^ = 0.0%, *P* = 0.645). The result of the publication bias analysis is presented in Fig. 3B. The funnel plot was symmetric, and the absence of publication bias was supported by both Egger’s and Begg’s tests (*P* > 0.05). Sensitivity analysis was performed to evaluate the stability of the combined effects of the two drugs, and the results are shown in Fig. 3C. After excluding one study each time and repeating the analysis, the pooled estimate for the rest of the studies was within the 95% CI (3.27, 6.90), indicating that the results were stable and reliable.

### Changes in clinical symptoms

**Fig. 4:**
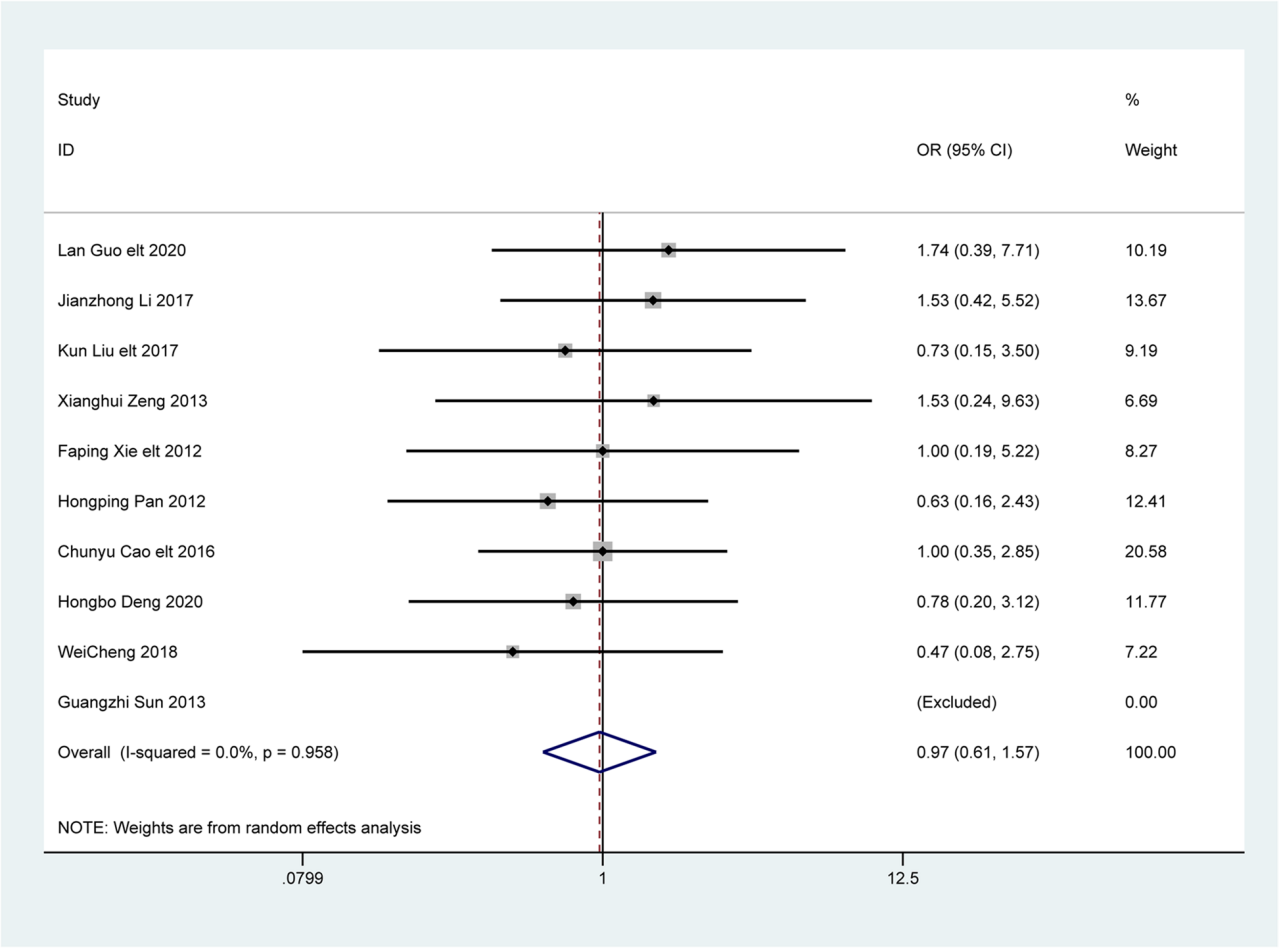
The combined effect results of clinical symptoms difficulty defecation in each study

**Fig. 5:**
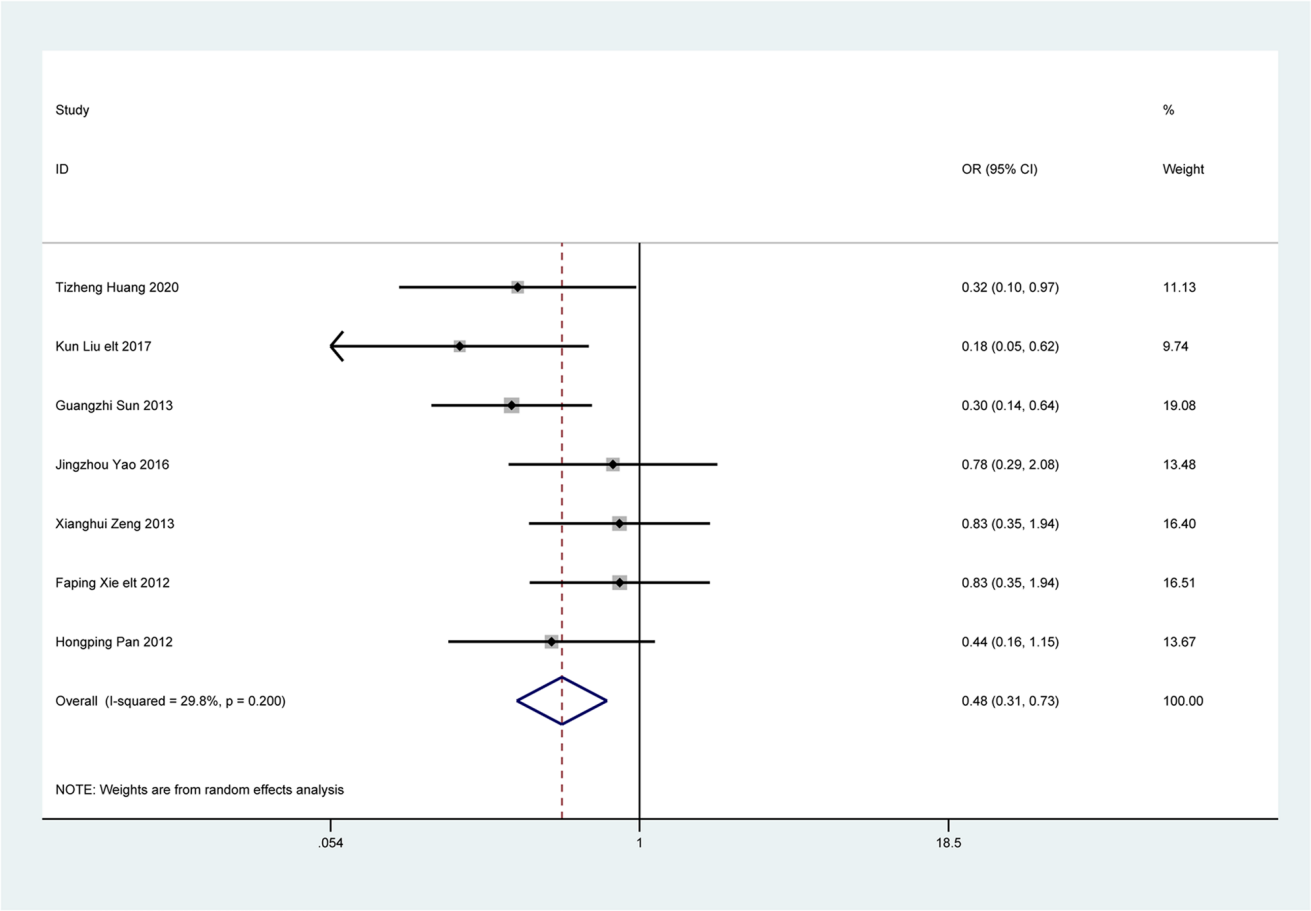
The combined effect results of clinical symptoms fecal character in each study

**Fig. 6:**
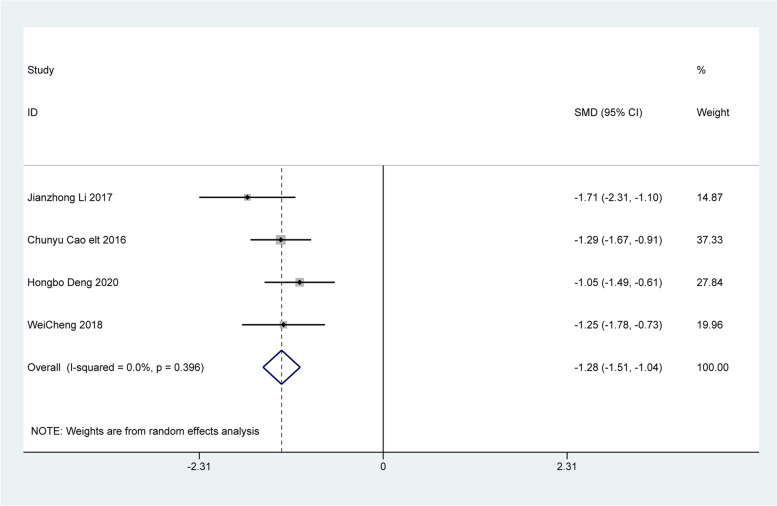
The combined effect results of clinical symptoms less defecation in each study

**Fig. 7:**
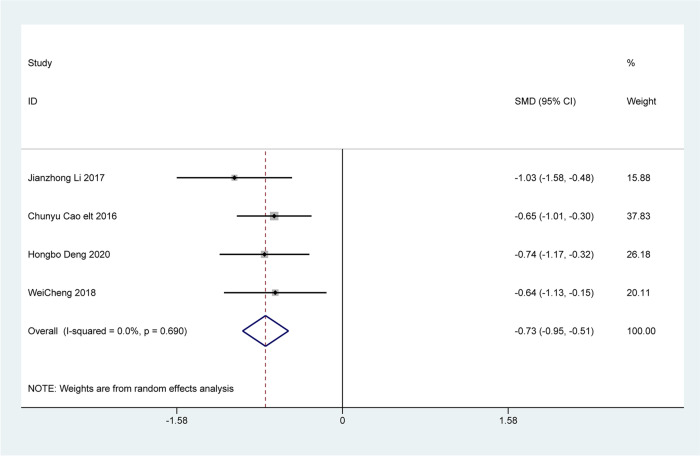
The combined effect results of clinical symptoms the defecation interval in each study

Four articles analyzed changes in clinical symptoms, including difficulty in defecation, fecal character, defecation frequency, and defecation interval. The analysis revealed that patients treated with Bifidobacterium quadruple viable tablets combined with mosapride citrate showed lower scores for defecation difficulty [SMD=-1.28, 95% CI (-1.51, -1.04), Z = 10.75, *P* < 0.001; I^2^ = 0.0%, *P* = 0.396, Fig. 4], fecal character [SMD=-0.73, 95% CI (-0.95, -0.51), Z = 6.57, *P* < 0.001; I^2^ = 0.0%, *P* = 0.690, Fig. 5], less defecation [SMD=-0.95, 95% CI (-1.17, -0.73), Z = 8.32, *P* < 0.001; I^2^ = 0.0%, *P* = 0.902), Fig. 6] and the defecation interval [SMD=-1.04, 95% CI (-1.26, -0.81), Z = 9.03, *P* < 0.001; I^2^ = 0.0%, *P* = 0.999, Fig. 7] than those treated with mosapride citrate alone. No heterogeneity was observed among the studies.

### Recurrence rate after treatment

**Fig. 8:**
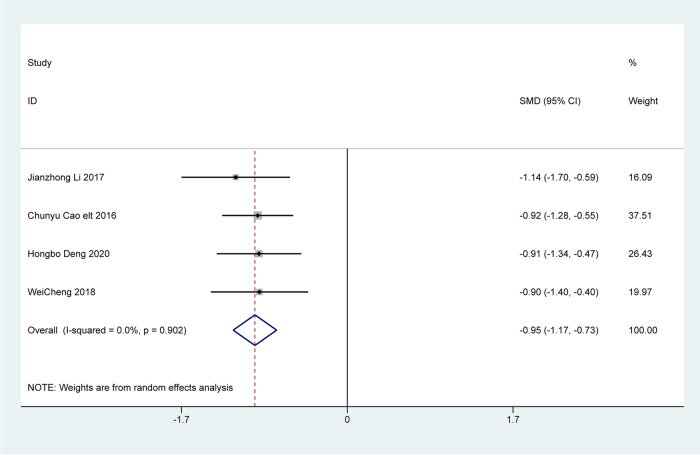
The combined effect results of Recurrence rate in each study

Seven articles analyzed the recurrence rate of constipation after treatment. The random effects model showed that the recurrence rate was significantly lower in patients treated with Bifidobacterium quadruple viable tablets combined with mosapride citrate compared to the control group, which showed statistically significant differences [OR = 0.48, 95% CI (0.31, 0.73), Z = 3.38, P = 0.001; I^2^ = 29.8%, *P* = 0.200, Fig. 8]. No heterogeneity was observed among the studies.

### Adverse reaction rates of treatments

**Fig. 9:**
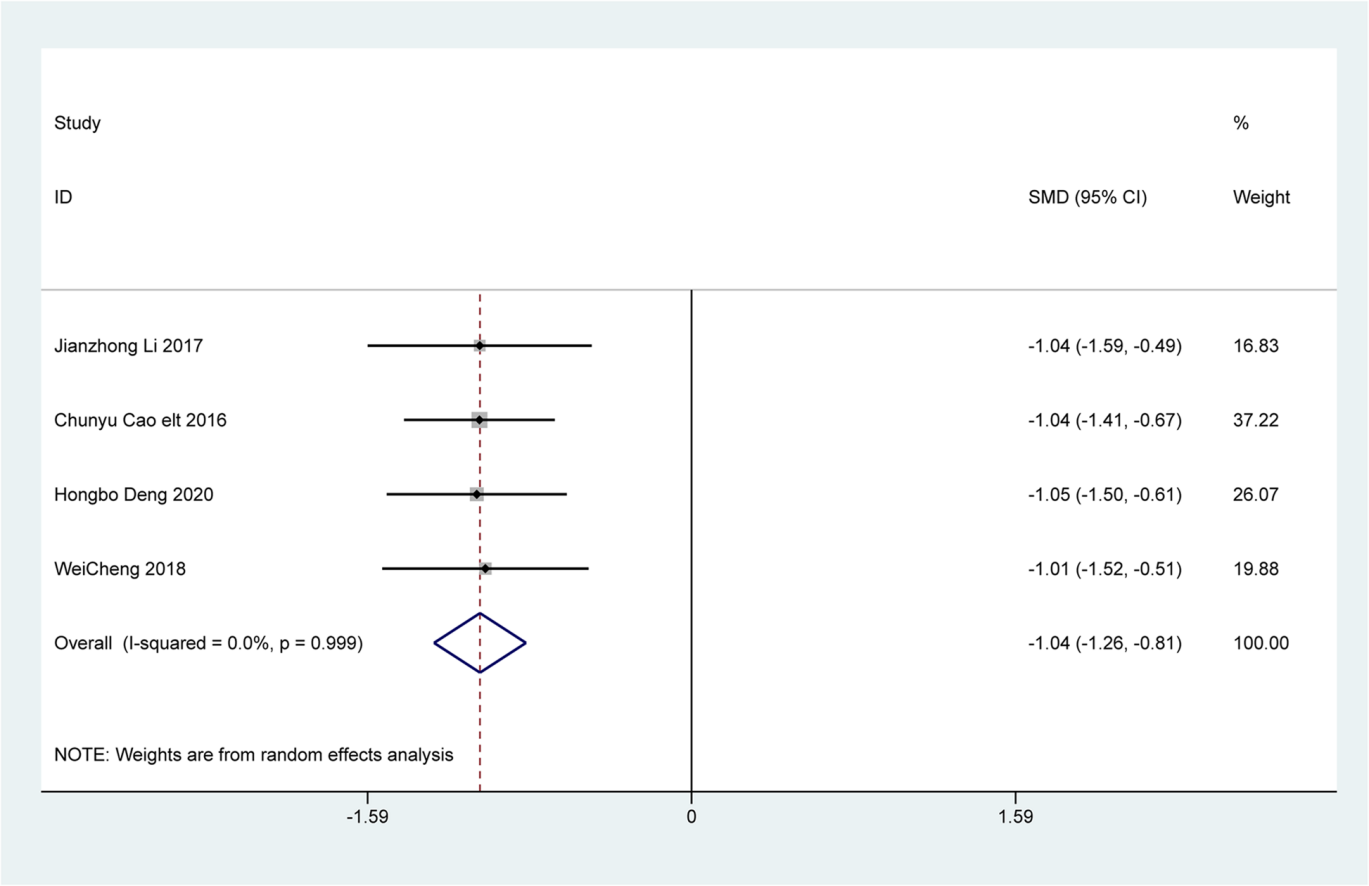
The combined effect results of Adverse reaction rate in each study

Ten articles were analyzed for adverse reaction rates. The results of the random effects model showed no significant difference in the incidence of adverse reactions between patients treated with Bifidobacterium quadruple viable tablets combined with mosapride citrate and that of the control group [OR = 0.97, 95% CI (0.61,1.57), Z = 0.11, P = 0.911; I^2^ = 0.0%, P = 0.958, Fig. 9]. No heterogeneity was observed among the studies.

## Discussion

FC is one of the most prevalent gastrointestinal disorders encountered in clinical practice [17]. Its etiology and pathophysiology are likely multifactorial. Current research suggests that patients with FC have imbalanced intestinal flora and significantly slower intestinal peristalsis [18, 19]. Recently, probiotics have emerged as adjuncts to normalize gut transit time and alleviate symptoms [19]. The effectiveness of probiotics in improving intestinal transit time is generally considered to be strain-specific [20]. Probiotics can be an effective treatment for irritable bowel syndrome, although the specific species and strains that provide the most benefits remain unclear [21]. Non-pharmacological interventions, such as probiotics, synbiotics, increased water intake, dry cupping, and additional biofeedback or behavioral therapy, have shown promise as effective and safe approaches to treat FC in children; however, further research is necessary to fully explore the utility of probiotics in alleviating FC symptoms in patients of all ages [22].

A recent systematic review and meta-analysis concluded that probiotics may improve overall intestinal transit time and defecation frequency in adults with FC, despite a large amount of heterogeneity among studies [23]. The consumption of probiotics, particularly multispecies probiotics, may lead to a notable reduction in gut transit time, increased stool frequency, and improved stool consistency [24]. In a randomized controlled trial, a significant improvement in average stool consistency was observed among participants receiving a probiotic compared to the placebo group after the first week of intervention. In the same study, patients receiving Bifidobacterium quadruple viable tablets exhibited lower fecal character scores than those in the control group [25]. In our meta-analysis, the random effects model indicated that the combination of Bifidobacterium quadruple viable tablets with mosapride citrate had a higher efficacy rate in treating constipation than mosapride citrate tablets alone.

As first-line treatments for FC, fiber and osmotic laxatives can increase stool frequency by an average of 1.4 total bowel movements per week [26]. Furthermore, a recent study demonstrated an increase in the frequency of bowel movements per week in individuals with constipation who received chicory inulin [27]. In our study, patients treated with Bifidobacterium quadruple viable tablets combined with mosapride citrate had lower defecation interval scores than those in the control group. Compared to the control group, patients who received the Bifidobacterium quadruple viable tablets exhibited lower defecation difficulty scores and lower fecal character scores. Colonization of germ-free rats with *L. acidophilus* and *Bifidobacterium* contributed to the normalization of intestinal transit [28, 29]. Similarly, the Bifidobacterium quadruple viable tablets used in the current study may have improved gut motility in patients who received this treatment. We hypothesize that the Bifidobacterium tablets play a protective role in preserving the integrity of the intestinal mucosa and contribute to its improved function.

Consistent with our findings, a meta-analysis demonstrated that probiotic (*Bifidobacterium* and *L. plantarum*) interventions were significantly superior to a placebo in the treatment of FC, showing improved efficacy and reducing the recurrence rate of constipation, thereby enhancing clinical effectiveness [23]. Regarding safety, the introduction of combined medication appeared to reduce adverse reactions in individuals who received this treatment compared to the control group. Our results did not indicate a significant difference in adverse effects between the two groups; however, this could be attributed to the relatively short follow-up period or the limited number of patients included in this study. This meta-analysis is subject to additional limitations. None of the studies included in this analysis employed double-blind methods, and geographical restrictions may affect the generalizability of the findings.

## Conclusion

The efficacy rate of Bifidobacterium quadruple viable tablets combined with mosapride citrate demonstrated higher efficacy in the treatment of patients with constipation compared to mosapride citrate alone. Furthermore, this treatment proved safe for managing constipation. The findings of this study highlight the potential of probiotics to positively influence gut health and microbial profiles in patients with FC.

## Supplementary Information

**Additional file 1.**

## Data Availability

The datasets used and/or analysed during the current study available from the corresponding author on reasonable request.

## Abbreviations

FC: Functional constipation
5-HT4: 5-hydroxytryptamine4
SMD: Standardized Mean Difference
